# Evaluation of Mitochondrial DNA (mtDNA) Copy Number Alterations and Clinical Parameter Correlations in Patients with Methamphetamine Use Disorder

**DOI:** 10.1192/j.eurpsy.2025.607

**Published:** 2025-08-26

**Authors:** H. M. Aytac, Y. Oyaci, M. Pehlivan, S. I. Azizoglu, O. Guclu, S. Pehlivan

**Affiliations:** 1 Institute of Graduate Studies in Health Sciences, Istanbul University; 2 Department of Psychiatry, Basaksehir Cam and Sakura City Hospital, University of Health Sciences; 3 Department of Medical Biology, Istanbul Faculty of Medicine, Istanbul University; 4 Department of Hematology, Basaksehir Cam and Sakura City Hospital, University of Health Sciences, Istanbul, Türkiye

## Abstract

**Introduction:**

Methamphetamine use disorder (MUD) and associated conditions impose a significant burden not only on affected individuals and their families but also on communities. Neurotransmitter system imbalances, mitochondrial dysfunction, oxidative stress, and activation of the inflammasome have all been implicated in methamphetamine-induced neurotoxicity. However, whether MUD is associated with peripheral mtDNA alterations remains uncertain.

**Objectives:**

This study aimed to investigate the relationship between mtDNA copy number and clinical parameters in individuals diagnosed with MUD, comparing them with healthy controls.

**Methods:**

Our study is a case-control research involving 52 patients diagnosed with MUD based on DSM-5 criteria and 52 healthy controls. Peripheral blood samples were collected, and leukocytes were isolated using the ELK Biotech Genomic DNA Extraction Kit for genomic DNA extraction. DNA samples were diluted to a concentration of 0.5-2 ng/μl for mtDNA copy number analysis, which was performed using the ScienCell qPCR Assay Kit. Two qPCR reactions were carried out for each sample using mtDNA and SCR primer sets. Known mtDNA copy number samples were used as references, and mtDNA copy numbers for each sample were calculated using the comparative ^∆∆^Cq method.

**Results:**

When comparing the mtDNA copy numbers between patients diagnosed with MUD and the control group, the mtDNA copy number in individuals with MUD was found to be significantly lower than that of the control group (p=.001). After controlling for age and gender, clinical parameters (suicide attempt, non-suicidal self-injury, duration of disorder, withdrawal symptoms, psychosis) and scale scores were compared based on mtDNA copy numbers. Among these, only substance use duration showed a statistically significant difference between the groups (p=.045). Additionally, in this study, a significant negative correlation was found between mtDNA copy numbers and the duration of disorder in MUD patients (r=-.369, p=.008).

**Conclusions:**

This is the first study examining the relationship between peripheral mtDNA alterations and clinical parameters in individuals diagnosed with MUD. A previous study showed that individuals with MUD exhibited decreased mtDNA copy numbers and increased mtDNA damage compared to healthy individuals in the Chinese population. Consistent with these findings, it was suggested that methamphetamine could lead to mitochondrial dysfunction and a reduction in mtDNA copy numbers in cell lines and animal models. In conclusion, individuals with MUD show decreased mtDNA copy numbers in peripheral blood samples, potentially related to increased autophagy. In this context, the reduction in mtDNA copy numbers could be used as a biomarker for MUD, and preventing this reduction may be beneficial in the clinical treatment of addiction.

**Disclosure of Interest:**

None Declared

